# Male patients require higher optimal effect-site concentrations of propofol during i-gel insertion with dexmedetomidine 0.5 μg/kg

**DOI:** 10.1186/s12871-016-0186-1

**Published:** 2016-03-22

**Authors:** Jung Ju Choi, Ji Young Kim, Dongchul Lee, Young Jin Chang, Noo Ree Cho, Hyun Jeong Kwak

**Affiliations:** 1 Department of Anesthesiology and Pain Medicine, Gachon University, Gil Medical Center, 1198 Guwol-dong, Namdong-gu, Incheon, 405-760 Republic of Korea; 2 Department of Anesthesiology and Pain Medicine, Anesthesiology and Pain Research Institute, Yonsei University College of Medicine, Seoul, South Korea

**Keywords:** Propofol, I-gel, Dexmedetomidine

## Abstract

**Background:**

The pharmacokinetics and pharmacodynamics of an anesthetic drug may be influenced by gender. The purpose of this study was to compare effect-site half maximal effective concentrations (EC50) of propofol in male and female patients during i-gel insertion with dexmedetomidine 0.5 μg/kg without muscle relaxants.

**Methods:**

Forty patients, aged 20–46 years of ASA physical status I or II, were allocated to one of two groups by gender (20 patients per group). After the infusion of dexmedetomidine 0.5 μg/kg over 2 min, anesthesia was induced with a pre-determined effect-site concentration of propofol by target controlled infusion. Effect-site EC50 values of propofol for successful i-gel insertion were determined using the modified Dixon’s up-and-down method.

**Results:**

Mean effect-site EC50 ± SD of propofol for successful i-gel insertion was significantly higher for men than women (5.46 ± 0.26 μg/ml vs. 3.82 ± 0.34 μg/ml, *p* < 0.01). The EC50 of propofol in men was approximately 40 % higher than in women. Using isotonic regression with a bootstrapping approach, the estimated EC50 (95 % confidence interval) of propofol was also higher in men [5.32 (4.45–6.20) μg/ml *vs.* 3.75 (3.05–4.43) μg/ml]. The estimated EC95 (95 % confidence interval) of propofol in men and women were 5.93 (4.72–6.88) μg/ml and 4.52 (3.02–5.70) μg/ml, respectively.

**Conclusions:**

During i-gel insertion with dexmedetomidine 0.5 μg/kg without muscle relaxant, male patients had higher effect-site EC50 for propofol using Schnider’s model. Based on the results of this study, patient gender should be considered when determining the optimal dose of propofol during supraglottic airway insertion.

**Trial registration:**

ClinicalTrials.gov identifier: NCT02268656. Registered August 26, 2014.

## Background

The i-gel airway is a single-use supra-glottic airway device. It was designed to fit peri-laryngeal structures and has an anatomically designed cuff filled with a medical grade thermoplastic elastomer gel. I-gel has been reported to have several potential advantages, such as, easier insertion and less tissue compression, over other supra-glottic airways with an inflatable cuff [1, 2].

Target controlled infusion (TCI) of propofol is widely used for supra-glottic airway insertion without neuromuscular blockade [3–5]. However, to obtain sufficient anesthetic depth to decrease airway reactivity, the effect-site concentration of propofol should be increased to a level that may lead to hypotension and bradycardia [3–5]. To avoid such complications, opioids or α_2_-agonistsis are concomitantly used as adjuvants during anesthesia induction [5–8]. Dexmedetomidine is a highly selective, short-acting α_2_-agonist with dose-dependent analgesic, sedative, and anxiolytic effects. Jang et al. [5] reported that preoperative dexmedetomidine infusion of 1 μg/kg decreased the effect-site half maximal effective concentration (EC50) of propofol by 53 % for successful i-gel insertion without muscle relaxants in male patients.

The pharmacokinetics and pharmacodynamics of anesthetic drugs may be influenced by gender [9], and previous studies have reported that male patients require significantly higher doses of propofol for loss of consciousness than female patients during anaesthetic induction [10, 11]. The purpose of this study was to compare effect-site EC50 values of propofol in male and female patients during i-gel insertion with dexmedetomidine 0.5 μg/kg without muscle relaxants. We hypothesized that male patients would require higher effect-site EC50 for successful i-gel insertion with dexmedetomidine pretreatment.

## Methods

This study was approved by the Institutional Review Board of Gil Medical Center (IRB no. GCIRB 2014–153) and registered at ClinicalTrials.gov (NCT 02268656). Written informed consent was obtained from all patients.

### Subjects

Forty patients aged 20–46 years of ASA physical status I or II undergoing minor surgery (duration of 1–2 h) were enrolled in the study. Patients were allocated to male or female groups (20 patients per group). Patients were excluded if they had a predicted difficult airway, a recent upper respiratory infection, unstable teeth, reactive airway disease, or were obese (body mass index > 30 kg/m^2^).

### Anesthesia and monitoring

Patients were not premedicated. After arrival in the operating theatre, electrocardiogram, pulse oximetry, automatic non-invasive blood pressure, and bispectral index (BIS) (BIS VISTA^TM^ monitor, four electrode sensor; Aspect Medical Systems, Norwood, MA, USA) were monitored. For pre-oxygenation, 100 % oxygen was administered for 3 min.

### Target effect site concentration of propofol

Before anesthetic induction, dexmedetomidine 0.5 μg/kg was infused for 2 min as described previously [12]. One minute later, lidocaine 30 mg injection was followed by propofol infusion using a TCI pump (Orchestra; Fresenius-Vial, Brezins, France) and Schnider’s pharmacokinetic model for propofol [13]. If necessary, ventilation was assisted manually to maintain an end-tidal CO_2_ tension between 30 and 35 mmHg. Five minutes after propofol infusion, an i-gel airway (Intersurgical Ltd, Wokingham, Berkshire, UK) was inserted after confirming target concentration and a BIS score below 60. One experienced anesthesiologist inserted an i-gel using a standard technique. After placing the patient in the sniffing position (head extended and neck flexed), the chin was gently pressed down and a lubricated i-gel was inserted gently towards the hard palate until resistance was felt. I-gel size was chosen according to patient weight; #3 for a weight of 30–60 kg and #4 for a weighing of 50–90 kg.

Target effect-site concentration of propofol for the first patient in each group was set at 5 μg/ml. Target effect-site concentrations of propofol for the next patients were determined by increasing or decreasing target effect-site concentrations (0.5 μg/ml as a step size) according to the response of the previous patient using the modified Dixon’s up and down sequential method [14]. Successful i-gel insertion was defined as proper movement of the chest and a continual end-tidal CO_2_ tension wave (without air leakage) at a peak airway pressure of <10 cmH_2_O. Insertion failure was defined as difficult mouth opening, gagging, coughing, laryngeal spasm, and signs of irritable body movements. In addition, if a patient showed an inadequate anesthetic level, such as, intact eyelid reflex or a high BIS score of above 60, insertion was regarded as having failed, and additional propofol was administered. The physician that conducted and evaluated insertion conditions was unaware of propofol effect-site concentration. A single assessment was obtained from each patient as whether insertion was successful or not. Only, the result of the first attempt was used for the analysis. After the assessment of ‘success’ or ‘failure’, the further anesthesia induction and maintenance were performed based on clinical need.

BIS score and hemodynamic data were measured at baseline (T0), after dexmedetomidine administration (T1), immediately before (T3) and 1 min after (T4) i-gel insertion. Bradycardia was defined as a decrease of > 30 % from baseline or a heart rate (HR) of < 45 beats/min persisting for more than 30 s and was treated with intravenous atropine 0.5 mg. Hypertension was defined as an increase of >20 % from baseline or a mean arterial pressure (MAP) of > 120 mmHg and was treated with intravenous nicardipine 300 μg.

According to Dixon’s up-and-down method, the stopping rule required at least six pairs of ‘success’ and ‘failure’ [14], and data from seven independent pairs of patients were collected for this study. The effect-site EC50 of propofol during i-gel insertion was defined as the mean of midpoint concentrations of all independent pairs of patients after seven crossover points were obtained in each group. The data were also subjected to isotonic regression analysis for calculations of EC50 and EC95 with the 95 % confidence intervals (CI) in each group [14]. An adjusted response probability was easily calculated by the pooled-adjacent-violators algorithm (PAVA) and the CI was estimated by a bootstrapping approach [15, 16]. If the value of EC50 did not overlap at the level of 83 % CI, the null hypothesis of equal concentration could be rejected as a type I error of 0.05 [17].

### Statistics

Results are reported as means ± standard deviations (SDs), median (ranges), or as numbers of patients. Statistical analysis was performed using the SPSS ver. 12.0 for Windows (SPSS Inc., Chicago, IL). Demographic data were analyzed using the independent *t*-test or the chi-square test as appropriate. Hemodynamic and BIS data were analyzed using repeated-measures ANOVA. Statistical significance was accepted for *p* values of < 0.05.

## Results

Twenty-patients were enrolled in each group. Patient characteristics and causes of i-gel insertion failure are listed in Table 1. Mean weight and height were significantly higher in the male group (all *p* values < 0.01), whereas group mean ages were similar. Insertion occurred in 10 men and 9 women, and causes of failure were not different in the two groups.

**Table 1:** Patients Characteristics

Variables	Male (*n =* 20)	Female (*n =* 20)
Age (years)	35 ± 11	35 ± 9
Weight (kg)	70 ± 10	57 ± 8^*^
Height (cm)	171 ± 6	159 ± 7^*^
ASA physical status (I/II)	18/2	19/1
Cause of failure	(*n =* 10)	(*n =* 9)
Difficult mouth opening	2	0
Gross purposeful movement	6	5
Gagging	8	5
Coughing	3	0
Laryngospasm	0	0
Sedation failure	2	2

Values are represented as mean ± SD or numbers of patients. ASA, American Society of Anesthesiologists. ^*^ *p* < 0.05, male vs. female

**Fig. 1:**
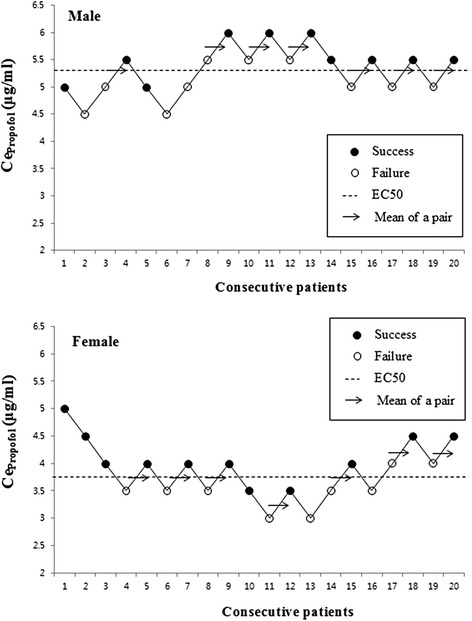
Consecutive effect-site concentration of propofol (Ce_propofol_) for i-gel insertion in male (upper) and female (lower) patients after preoperative dexmedetomidine (0.5 μg/ml) administration. Horizontal lines represent the half maximal effective effect-site concentrations (EC50). Mean EC50 (± SD) values in the male and female groups were 5.46 ± 0.26 and 3.82 ± 0.34 μg/ml, respectively (*p* < 0.01)

**Fig. 2:**
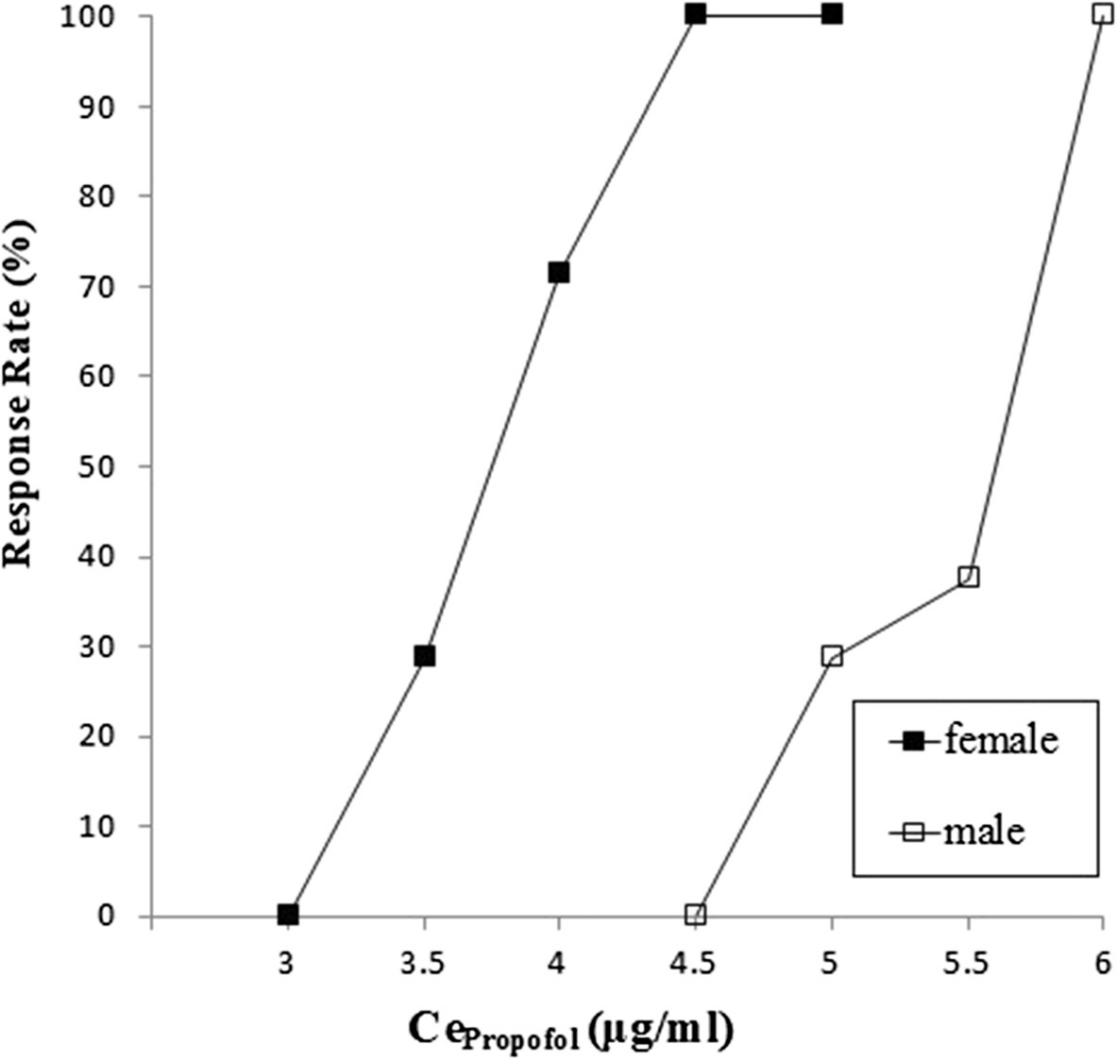
Pooled-adjacent-violators algorithm (PAVA) response rate in male (unfilled square) and female (filled square) patients. EC50 (95 % CI) values in male and female groups were 5.32 (4.45–6.20) and 3.75 (3.05–4.43) μg/ml, respectively, and corresponding EC95 (95 % CI) values were 5.93 (4.72–6.88) and 4.52 (3.02–5.70) μg/ml, respectively. Isotonic regression showed the EC50 value was significantly higher in the male group than in the female group

The up-and-down sequences in consecutive patients are illustrated in Fig. 1. According to modified Dixon’s up-and-down method, the effect-site EC50 (± SD) of propofol for successful i-gel insertion was significantly higher for men than women (5.46 ± 0.26 *vs*. 3.82 ± 0.34 μg/ml, *p* < 0.01). By isotonic regression with bootstrapping (Fig. 2), the estimated effect-site EC50 (83 % CI, 95 % CI) of propofol was also higher in men [5.32 (4.60–6.02, 4.45–6.20) *vs.* 3.75 (3.18–4.31, 3.05–4.43) μg/ml]. The effect-site EC95 values (95 % CI) of propofol in the male and female groups were 5.93 (4.72–6.88) μg/ml and 4.52 (3.02–5.70) μg/ml, respectively. The actual mean doses infused over 5 min (from start of the infusion to the completion of i-gel insertion) in men and women were 3.35 ± 0.81 mg/kg and 2.77 ± 0.41 mg/kg, respectively, and thus, actual mean dose was also higher for men (*p* = 0.008).

BIS and hemodynamic data for successful i-gel insertion are listed in Table 2. No significant intergroup difference in BIS, MAP, or HR was observed at any time (all *p* values > 0.05). BIS decreased significantly in both groups (both *p* < 0.01), but these change were similar in the two groups (*p* = 0.969). Similarly, MAP and HR changed significantly (both *p* < 0.001), and these changes were also similar in the two groups (*p* = 0.35 and 0.95, respectively). No laryngospasm or desaturation (defined as SpO_2_ < 90 %) occurred during the study. After dexmedetomidine administration, one male patient had bradycardia, which was treated with atropine, and one female had hypertension, which was treated with nicardipine.

**Table 2:** Hemodynamic and bispectral index data for successful i-gel insertion

	Group	T0	T1	T2	T3
BIS	Male (*n =* 10)	95 ± 3	92 ± 5	44 ± 13	43 ± 17
	Female (*n =* 11)	94 ± 5	94 ± 5	43 ± 15	44 ± 14
MAP	Male (*n =* 10)	100 ± 17	106 ± 15	90 ± 12	88 ± 10
	Female (*n =* 11)	102 ± 18	117 ± 22	91 ± 12	95 ± 13
HR	Male (*n =* 10)	67 ± 14	58 ± 8	60 ± 14	64 ± 13
	Female (*n =* 11)	69 ± 13	61 ± 12	57 ± 10	61 ± 11

Values are represented as mean ± SD. BIS, bispectral index; MAP, mean arterial blood pressure (mmHg); HR, heart rate (beats/min). T0, baseline value (before anesthesia induction); T1, after dexmedetomidine administration; T2, immediately before i-gel insertion; T3, 3 min after i-gel insertion

## Discussion

This study demonstrates that during i-gel insertion with dexmedetomidine pretreatment, male patients require significantly higher effect-site EC50 values of propofol using Schnider’s model. The effect-site EC50 values of propofol for successful i-gel insertion with dexmedetomidine 0.5 μg/kg without muscle relaxants in male and female patients were found to be 5.46 and 3.82 μg/ml, respectively.

There is growing interest in gender difference from the pharmacokinetic and pharmacodynamic perspectives. In the case of propofol, male patients have been reported to have significantly longer recovery times than female patients [18, 19], which supports the possibility that women are less sensitive to propofol. However, for loss of consciousness at induction, propofol requirements were found to be significantly greater for men [10, 11]. A recent study by Fu et al. [20] reported the menstrual cycle may influence EC50 for loss of consciousness and that it was significantly lower in the luteal phase than in the follicular phase. The authors concluded that anesthetic effects might be influenced by difference in progesterone levels [20]. To the best of our knowledge, no study has previously reported a gender effect on the EC50 of propofol with respect to supraglottic airway insertion. The present study shows a gender difference in effect-site EC50 of propofol for i-gel insertion by the modified Dixon’s method and isotonic regression analysis. Taken together, a female sex hormone-induced gender difference in the pharmacodynamics of propofol provides a possible explanation for our results.

Another possible explanation is that the suppression of airway reactivity or cough reflex by anesthetics exhibits a gender difference, because this suppression is one of the most important aspects of achieving successful supraglottic airway insertion without a neuromuscular blocking agent. In an earlier study about remifentanil requirements for cough suppression during emergence, it was reported that its antitussive effect was achieved at a significantly higher concentration in males than in females [21]. The authors suggested that during anesthesia emergence anesthetic concentrations required for cough suppression may differ for genders under similar clinical conditions. In an experimental study, it was shown male sex hormones might promote reflex airway responsiveness and that there was a gender disparity in terms of airway responsiveness to cholinergic stimulation [22].

Opioid analgesics are frequently used adjuncts during propofol anesthesia, and have been reported to exhibit gender dependent pharmacodynamic properties [9]. However, no report has been issued on such dependencies for dexmedetomidine. It has been reported the pharmacokinetics of dexmedetomidine are best described by a three-compartment model, and that the addition of age, weight, lean body mass, and body surface area do not improve the predictive value of the model [23]. In addition, for anesthetic interaction producing hypnosis and immobility, propofol and opioids appear to act synergistically, whereas propofol and clonidine seem to act additively [24]. Thus, we selected dexmedetomidine as an anesthetic adjuvant, and believe it is unlikely to have influenced the gender difference observed in the present study. The preoperative dexmedetomidine dose of 0.5 μg/kg was chosen based on the results of a previous study, which reported that the effective dose of dexmedetomidine in 50 % patients was 0.55 μg/kg during anesthetic induction with propofol 2 mg/kg [12]. As for the propofol dose sparing effect, a recent study reported that dexmedetomidine 0.5ug/kg reduced the propofol dose required for anesthesia induction [25]. Further studies are needed to elucidate the nature of the pharmacodynamic interaction between propofol and dexmedetomidine and its dependence on gender.

In the present study, propofol was infused using a TCI pump (Orchestra; Fresenius-Vial, Brezins, France) using Schnider’s pharmacokinetic model for propofol. Several pharmacokinetic models are used for propofol, and the most widely used are the Marsh, modified Marsh, and Schnider models. Had a pharmacokinetic model other than the Schnider model been applied in the present study, undoubtedly different results would have been obtained. It has been reported that when the Marsh model is used, a high initial dose is required for anesthesia induction and that this causes overshooting, which results in a shorter loss of consciousness time and a lower estimated effect-site propofol concentration than when the Schnider model is used [26, 27]. In a previous study that applied the Marsh model, actual measured propofol concentration was 40 % higher than estimated concentration in men, but only a difference of 2 % was observed in women, so the estimate was less accurate in men [28]. However, the Schnider model makes a correction for the effect of body mass index, age and sex. The fact that Schnider model already incorporates a gender difference may make the result of study questionable. However, the gender difference taken into account in Schnider model is not likely to affect the result of this study since this difference in clearance based on lean body mass is important when propofol is infused during maintenance of anesthesia. This study encompass the induction period, which concerns the sensitivity to loss of consciousness, not the clearance of the drug from the system.

In this study, the optimal male and female doses were analyzed using EC50 values determined using Dixon’s up and down method, and because EC95 values are clinically significant, they were calculated by isotonic regression analysis. In the event, effect-site EC95 values (95 % CI) of propofol in the male and female groups were found to be 5.93 (4.72–6.88) μg/ml and 4.52 (3.02–5.70) μg/ml, respectively, which were not significantly different. We chose isotonic regression because progbit analysis is a parametric technique. In addition, isotonic regression has a smaller bias and tighter CIs compared to standard probit analysis [15].

The present study has several limitations that warrant consideration. First, we did not measure propofol plasma concentrations, but rather predicted propofol concentrations using Schnider’s pharmacokinetic model [13], in which time to peak effect and *k*_e0_ are 1.7 min and 0.46/min, respectively [29]. To ensure equilibration between blood- and effect-site concentrations, the steady-state infusion for propofol should be maintained for 15 min before giving a verbal command or administering other drugs [30]. However, many studies on supraglottic airway insertion have used loss of consciousness (LOC) time to enable prompt progress of surgery [31, 32]. In a previous study, LOC time of 171.4 ± 70.9 s was reported when 4 μg/ml of propofol was administered [33], and in another study, in which an effective site target concentration of 6 μg/ml of propofol was set, an induction time (defined from propofol infusion commencement to the completion of LMA insertion) of 146.9 ± 42.3 s was reported [31]. Based on these reports, the present study was conducted based on the assumption that 5 min of infusion would induce LOC. Second, dexmedetomidine preceded propofol only by about 3 min to minimize induction time, and since dexmedetomidine takes 15 min to achieve optimal effect, the EC50 of propofol may have been different had a 15 min time lag been used between dexmedetomidine and propofol. However, the effect of this is in our opinions unlikely to influence the observed gender-dependent dose difference. Third, this study was not conducted using a double blind design because the researcher that inserted the i-gel was aware of group identities. The researcher was only blinded to the concentration of propofol but the information of a previous patient may have affected the decision of the i-gel insertion status of the next patient. Fourth, weights obvious differed in the two study groups, and collinearity problems arise when an explanatory variable (covariate), in this case weight, is not independent. However, since we used a TCI pump and the Schnider model, which takes height, weight, and lean body mass into account as covariates, we believe the effect of weight difference was slight. Lastly, the menstrual cycle was not checked in the female group. Since female sex hormones may influence hypnotic drug dosage, further study is needed to elucidate the effect of the menstrual cycle on the EC50 of propofol.

## Conclusion

During i-gel insertion with dexmedetomidine 0.5 μg/kg without muscle relaxant, male patients were found to require a higher effect-site EC50 of propofol than female patients using Schnider’s model. Based on the results of this study, patient gender should be considered when determining the optimal dose of propofol required for supraglottic airway insertion.
